# Publication Rates of Podium Presentations at an Annual Orthopedic Surgery Resident and Fellow Research Symposium

**DOI:** 10.7759/cureus.57121

**Published:** 2024-03-28

**Authors:** Andrew George, Shari R Liberman, Bradley K Weiner, Kevin E Varner, Patrick C McCulloch, Robert A Jack, Timothy S Brown, Joshua D Harris

**Affiliations:** 1 Orthopedics & Sports Medicine, Houston Methodist Hospital, Houston, USA

**Keywords:** research symposium, publication rate, curriculum, orthopedics, resident education

## Abstract

Introduction

Research is an important aspect of residency and fellowship programs across the country. Developing strategies to foster research productivity is worthwhile. An annual research project is one strategy that some programs implement.

Methods

All resident and fellow (Sports Medicine, Adult Reconstruction, Spine) presentations at an orthopedic surgery department’s annual research symposium from June 2016 through June 2021 were identified. Abstract titles, title keywords, and author names were searched in PubMed and Google Scholar to identify the presence of a peer-reviewed publication. Using the total number of research symposium presentations given, the publication rate was calculated for each year, as well as collectively for 2016 to 2021. In addition to publication rate, first author percent, number of citations, Altmetric score, and journal impact factor were recorded. Current PGY-2 through PGY-5 residents completed a survey to assess the perceived value of the annual research symposium.

Results

Ninety-eight research symposium presentations were reviewed (69 residents, 29 fellows). Forty (58%) resident studies were published and 28 were first-author publications (70%). Thirteen (45%) fellow studies were published and seven were first-author publications (54%). Combining residents and fellows, the overall publication rate was 54% (53/98), and 66% of these (35/53) were first-author publications. There was a wide range of published manuscript journal impact factors, Altmetric scores, and number of citations. All residents surveyed reported finding value in the research symposium.

Conclusion

The overall publication rate of presentations at an annual orthopedic surgery department research symposium between 2016 and 2021 was 54%, consistent with publication rates reported at National Orthopedic Surgery Society meetings. All residents reported finding value in the annual research symposium. The results of this study support the academic value of implementing a required annual research project and may provide a useful gauge to inform residency and fellowship curricula at other institutions.

## Introduction

Research is a core element of orthopedic surgery residency and fellowship programs, as well as orthopedic surgery departments across the country. Accordingly, developing strategies to incorporate research into a residency or fellowship curriculum is a key priority for many programs. Several training programs offer dedicated time during residency for research, with some offering an optional or required extra year of residency specifically for research. Some programs implement an annual research project in which residents or fellows present their work at a department-wide symposium. While this strategy should theoretically increase research productivity among trainees and the department, little research has reported the ultimate publication percentage of these presentations.

Publication rates of presentations and abstracts at annual orthopedic meetings have been extensively reported in the literature. Publication rate is an important metric because it can help quantify the academic value of a meeting, specifically in regards to knowledge dissemination and the overall impact on the field of orthopedic surgery. Over the past two decades, the publication rate has varied from 40% to 70% [1]. At the annual American Academy of Orthopaedic Surgeons (AAOS) meeting, the publication rate increased from 49% in 2001 to 61% in 2010 and increased further to 69.9% between 2014 and 2017 [1-3]. The publication rates at orthopedic subspecialty meetings have also been reported in the literature, and on average, tend to be around 60%.

The purpose of this study was to assess the publication rates of presentations at an orthopedic surgery department’s annual resident and fellow research symposium. We specifically sought to evaluate the value from an academic output standpoint, as well as the perceived value from a resident standpoint. The hypothesis was that publication rates would be similar to those reported at annual national orthopedic meetings.

## Materials and methods

In this retrospective study, 98 presentations (69 by residents and 29 by fellows) at a single orthopedic surgery department’s annual research symposium (held yearly in June) from 2016 through 2021 were identified using the annual research symposium abstract booklets. The analyzed orthopedic surgery residency program has three residents per class. The fellowship programs analyzed in this investigation included Sports Medicine (three fellows per class), Adult Reconstruction (three fellows per class), and Spine (two fellows per class). The year 2020 was excluded, as the research symposium was canceled due to the COVID-19 pandemic. The review was conducted in July 2023, allowing for a total of five years with a minimum two-year follow-up. The search strategy was adapted by methods used in similar studies evaluating annual meetings, notably by Donegan et al. and Le et al. [1,2]. Each abstract title, as well as title keywords, were searched in PubMed and Google Scholar to identify the presence of a peer-reviewed publication. If no results were found, these databases were searched using both abstract titles and author names. If this also yielded no results, the resident’s or fellow’s full individual publication list was queried to identify any potentially missed publications. For all publications identified, the methods, results, and conclusions were also evaluated to ensure they were the same studies as reported in the research symposium abstracts.

For each publication identified, the resident or fellow author position was noted (first author, last/senior author, middle author). Publications were excluded if the presenting resident or fellow was not listed as an author. The total number of residents and fellows listed as authors on each publication was recorded. The time (in months) between the research presentation and publication was also recorded. To assess the publication’s scholarly impact, the journal’s Impact Factor was collected using Journal Citation Reports [4] and recorded, and the Altmetric score [5] was collected and recorded (on 07/21/23). Additionally, the number of citations of each publication was recorded using Google Scholar. Current PGY2-5 residents completed a survey to assess the perceived value of the research symposium. First-year residents were excluded, as they had not yet participated in a research symposium.

Using the total number of research symposium presentations during the eligibility period, the publication rate was calculated for each year, as well as collectively for 2016 to 2021. Among published articles, the percentage of first-author publications was also calculated. Publication rates were calculated for residents alone, fellows alone, and residents and fellows combined. Publication rates were compared between residents and fellows, junior residents (PGY1-3) and senior residents (PGY4-5), and male and female trainees. Fisher’s exact test was performed to assess for differences between groups with a p-value less than 0.05 indicating statistical significance.

## Results

Of the 69 resident presentations, 40 studies were published (58%, Table 1) at an average of 12 months after the presentation. Of these 40, the presenting resident was the first author in 28 publications (70%). Of the 29 fellow presentations, 13 studies were published (45%, Table 2) at an average of 32 months after the presentation. Seven of these were first-author publications (54%). There was no significant difference in resident and fellow publication rates (p = 0.27). Combining residents and fellows, the overall publication rate was 54% (53/98), and 66% of these (35/53) were first-author publications. The combined average time to publication was 17 months. There were 83 total resident and fellow authors among the 53 publications (1.6 per publication).

**Table 1:** Resident publication rates and metrics * One duplicate excluded (one published study was presented in two different years) SD: standard deviation

Year	2016	2017	2018	2019	2021	Total*
Number of Symposium Abstracts	12	14	14	15	15	69
Number of Publications	8	11	9	8	5	40
Publication Percent (%)	67	79	64	53	33	58
First Author Percent (%)	75	73	100	75	0	70
Altmetric Score (mean +/- SD)	5.5 +/- 7.1	11.6 +/- 18.7	9.4 +/- 16.4	7.1 +/- 13.2	94 +/- 208.9	19.7 +/- 74.9
Impact Factor (mean +/- SD)	2.7 +/- 1.4	3.0 +/- 1.2	2.9 +/- 1.6	2.0 +/- 1.1	1.5 +/- 1.3	2.5 +/- 1.4
Citations (mean +/- SD)	47.0 +/- 57.6	17.5 +/- 15.4	24.8 +/- 20.8	6.2 +/- 4.7	1.4 +/- 1.5	20.7 +/- 31.6

**Table 2:** Fellow publication rates and metrics * One duplicate excluded (one published study was presented in two different years) SD: standard deviation

Year	2016	2017	2018	2019	2021	Total*
Number of Symposium Abstracts	5	5	7	7	6	29
Number of Publications	4	2	2	5	1	13
Publication Percent (%)	80	40	29	71	17	45
First Author Percent (%)	50	50	50	60	0	54
Altmetric Score (mean +/- SD)	15.8 +/- 19.3	3.5 +/- 3.5	4.0 +/- 4.2	0.8 +/- 1.5	95.0+/- 0.0	13.5 +/- 27.2
Impact Factor (mean +/- SD)	3.5 +/- 5.0	6.6 +/ 4.8	4.7 +/- 0.8	1.2 +/- 0.8	3.5 +/- 0.0	4.1 +/- 3.1
Citations (mean +/- SD)	23.5 +/- 18.1	11.0 +/- 5.7	5.5 +/- 6.4	0.6 +/- 0.9	0.0 +/- 0.0	9.3 +/- 13.5

The publication rates were generally consistent between 2016 and 2019, but there was a notable decrease in the final year, 2021. In 2021, the resident publication rate was 33% and the fellow publication rate was 17%. Of the studies published this year, there were no residents or fellows who were first authors. Of the 69 residents who presented at the research symposium between 2016 and 2021, 51 were male, and 18 were female. The male resident publication rate was 65% (33/51) and the female publication rate was 39% (7/18) (p = 0.09). Only 2 of the 29 fellows were female, limiting any meaningful analysis of gender differences in the fellow cohort. The overall publication rate of PGY-4 and PGY-5 residents was 71% compared with 50% in the PGY 1-3 classes (p = 0.09). There was a wide range of journal impact factors, Altmetric scores, and number of citations (Tables 1, 2). Looking at the senior author, the same senior author was present in 35% of resident publications (14/40). Aside from this, no other notable trends were looking at senior authors for either resident or fellow publications.

Current PGY 2-5 residents reported finding value in the research symposium, with 5/12 residents reporting it to be very valuable and 2/12 extremely valuable. Ten of the 12 residents surveyed noted the implementation of an annual research symposium allowed them to be more productive from a research standpoint. Ten of the 12 residents also reported they would be likely to recommend the implementation of an annual research symposium for other residency programs.

## Discussion

The purpose of this study was to assess the publication rates of presentations at an orthopedic surgery department’s annual resident and fellow research symposium. The hypothesis was supported, as the overall publication rate was similar to those reported at annual national orthopedic meetings over the last several years. Furthermore, two-thirds of the publications in this investigation were resident or fellow first-author publications, suggesting the majority of residents and fellows took an active role in their studies, as well as in writing manuscripts.

As research productivity is a priority for many orthopedic surgery residency and fellowship programs, developing strategies to promote engagement in meaningful research is worthwhile. While the implementation of an annual research project has its theoretical benefits, it comes with the potential cost of time spent away from other learning environments and opportunities such as the operating room, clinic, studying, innovation, administration, leadership, and community, among others. Therefore, assessing the true benefit of a research project is valuable. While several studies have looked at the publication rate of presentations and abstracts at national orthopedic meetings, this is the first study in the literature to report the publication rate of an annual presentation in an orthopedic surgery department.

In this study, the overall publication rate was 54%, consistent with the publication rates reported at national meetings. Donegan et al., Williams et al., and Le et al. evaluated the publication rates of presentations at the annual AAOS meeting in 2001, 2010, and 2014-2017, respectively [1-3]. The publication rate ranged from 49% in 2001 to 70% in 2014-2017. The publication rates at subspecialty meetings have also been reported in the literature, and on average, tend to be around 60% (Table 3).

**Table 3:** Orthopedic Surgery Society annual meeting publication rates AAOS: American Academy of Orthopaedic Surgeons, AOSSM: American Orthopaedic Society for Sports Medicine, AANA: Arthroscopy Association of North America, ASES: American Shoulder and Elbow Surgeons, AAHKS: American Association of Hip and Knee Surgeons, AOFAS: American Orthopaedic Foot & Ankle Society, NASS: North American Spine Society, POSNA: Pediatric Orthopaedic Society of North America [1,6-12]

Annual Meeting	Years	Publication Percentage
AAOS	2014-2017	70 [1]
AOSSM	2011-2015	45 [6]
AANA	2008-2012	59 [7]
ASES	2008-2012	61 [8]
AAHKS	2011-2015	60 [9]
AOFAS	2008-2012	62 [10]
NASS	2010-2012	44 [11]
POSNA	2013-2016	58 [12]

The publication rate and first author percentage were higher among residents compared with fellows (58% vs. 45% publication rate, 70% vs. 54% first author), but these were not significantly different. While this could be a beta error, and a larger sample size may detect a possible difference between these cohorts, the observed difference may be influenced by a drop in fellow involvement in their research projects once they graduate and move on to practice. Another factor could be increased resident motivation to publish in order to become a more competitive applicant to fellowship programs. Of note, the majority of residents surveyed reported the implementation of an annual research symposium allowed them to be more productive from a research standpoint. While annual research projects are generally more common in orthopedic fellowship programs (required in some fellowships) compared to residency programs across the country, the results of this study indicate there may be similar benefits in residency, at least in terms of ultimate research output. Among residents, senior residents (PGY-4 and 5) had a higher publication rate compared to PGY1-3 junior residents (71% vs. 50%). Although this difference was not significant, this too could be an underpowered comparison, and several factors play a role including senior residents having more time to work on research or potentially senior residents having more experience in effectively publishing their work.

This study has limitations. First, the study was conducted at a single institution, and the results may not be generalizable to other institutions. The ultimate publication rate is likely influenced by multiple factors, such as research infrastructure, faculty support, and resident/fellow curriculum, which all vary from institution to institution. Nonetheless, the results of this study may provide a useful gauge to inform residency and fellowship curricula at other institutions. Future work should look at larger data sets across multiple institutions to better account for these other factors, as well as more robustly compare differences between subsets of these populations. Second, the search was restricted to PubMed and Google Scholar using abstract titles and author names. As a result, there is a possibility some publications were not identified and included. However, the above methodology is standard in the existing literature evaluating publication rates at national orthopedic meetings, and previous work in this area has concluded the addition of other databases did not result in an increase in the percentage of published articles found [1]. Similarly, while this study had a two-year minimum follow-up, it is still possible some presentations included will be published in the next several years, so our reported publication rate could be an underestimation.

Finally, greater quantity is not necessarily a good thing. The authors strongly emphasize the role of high-quality research with proper development, study design, meticulous and ethical conduct, transparent and complete reporting, and succinct and well-written manuscripts. Many trainees feel the number of publications is the most important metric on a fellowship or job application. This is erroneous on both counts [13-15]; hence, the current investigation’s inclusion of Impact Factor, Altmetric scores, and number of citations in determining the academic influence of the paper.

## Conclusions

The overall publication rate of presentations at an annual orthopedic surgery department research symposium between 2016 and 2021 was 54%, consistent with publication rates reported at National Orthopedic Surgery Society meetings. All residents reported finding value in the annual research symposium. The results of this study support the academic value of implementing a required annual research project and may provide a useful gauge to inform residency and fellowship curricula at other institutions.
